# Association between a low IgE response to Phl p 5 and absence of asthma in patients with grass pollen allergy

**DOI:** 10.1186/1476-7961-11-3

**Published:** 2013-12-05

**Authors:** Eleonora Savi, Silvia Peveri, Cristoforo Incorvaia, Ilaria Dell’Albani, Francesco Marcucci, Giuseppe Di Cara, Franco Frati

**Affiliations:** 1 Allergy Unit, G. Da Saliceto Hospital, AUSL Piacenza, Italy; 2 Allergy/Pulmonary rehabilitation, ICP Hospital, Milan, Italy; 3 Medical and Scientific Department, Stallergenes, Viale Certosa 2, Milan 20155, Italy; 4 Spin-Off ATRP Srl, Allergic Tests Research and Production, Perugia, Italy; 5 Institute of Pediatrics, Department of Medical and Surgical specialty and Public Health, Perugia, Italy

**Keywords:** Asthma, Component-resolved diagnosis, Grass pollen, Phl p 5, Primary sensitizing allergens

## Abstract

**Background:**

The introduction of component-resolved diagnosis was a great advance in diagnosis of allergy. In particular, molecular allergy techniques allowed investigation of the association between given molecular profiles and clinical expression of allergy. We evaluated the possible correlation between the level of specific IgE (sIgE) to single components of *Phleum pratense* and clinical issues such as the severity of allergic rhinitis (AR) and the presence or absence of asthma.

**Methods:**

The study included 140 patients with rhinitis and/or asthma caused by sensitization to grass pollen. sIgE to Phl p 1, Phl p 5, Phl p 7, and Phl p 12 from *Phleum pratense* were measured, and the correlation between the stage of AR according to Allergic Rhinitis and its Impact on Asthma (ARIA) guidelines and the presence of asthma was studied by multivariate logistic regression in terms of sIgE and ARIA stage, while univariate logistic regression was used for IgE and a dichotomic classification of asthma as present or absent.

**Results:**

Ten patients had intermittent AR, 48 had mild persistent AR, and 82 had severe persistent AR. Asthma was present in 86 patients and absent in 54. A significant correlation was found between severe persistent AR and presence of asthma (*p* < 0.01). The only significant correlation between clinical data and sIgE values was that of low values of sIgE to Phl p 5 and absence of asthma (*p* < 0.01).

**Conclusions:**

This preliminary finding suggests that low values of sIgE to Phl p 5 are correlated with the absence of asthma in patients with grass-pollen induced allergy. The data, provided they are confirmed by further studies, could be useful when selecting patients who are candidates for allergen immunotherapy, since a higher risk of asthma could be used as a selection criterion for using this approach.

## Background

Until recently, the diagnosis of allergy has been based on the combination of data from clinical history and the results of skin tests and in vitro IgE tests. The availability of single allergen molecules for use instead of whole allergen sources has allowed a significant advance in the diagnostic workup of allergy [1,2]. In fact, this diagnostic approach, currently known as component-resolved diagnosis (CRD) [3], allows identification of the individual profile of IgE reactivity to the different molecules. The knowledge of the biological function of each molecule makes it possible to define its clinical importance in patients’ symptoms, distinguishing the genuine sensitizers from the molecules acting as simple cross-reacting components, with little clinical effect [4,5]. In grass pollen, a cause of respiratory allergy, a number of molecules has been identified and evaluated based on their clinical importance. The grass species commonly used as a model is *Phleum pratense*, which contains 13 different groups according to physicochemical and immunologic properties [6]. Of these groups, Phl p 4, Phl p 7, Phl p 11, and Phl p 12 are cross-reacting allergens that are not grass-specific, while Phl 1 and Phl p 5 are grass-specific. Among the cross-reacting allergens, Phl p 4 is a cross-reactive carbohydrate determinant (CCD)-bearing protein recently characterized in vitro [7], but which has an undefined clinical role; Phl p 11 cross-reacts with Ole e 1 from olive pollen [8], Phl p 7 and Phl p 12 are panallergens. Phl p 1, along with its homologues in other grass species, includes acidic glycoproteins, while Phl p 5 includes proteins with ribonuclease activity, presenting two non-glycosilated isoforms. Recent studies addressed the response to the various components of grass pollen in patients treated with allergen immunotherapy (AIT), in both subcutaneous and sublingual administration form, but reported different observations [9-13].

We here evaluated the possible correlation between the level of specific IgE (sIgE) to the single components of *P. pratense* and the clinical expression of allergy to grass pollen, namely the severity of allergic rhinitis (AR) and the presence or absence of asthma, in a group of patients with grass pollen-induced respiratory allergy.

## Methods

The study population consisted of 140 patients with rhinitis and/or asthma caused by sensitization to grass pollen, as assessed by positive skin prick tests (SPT) and a clear correlation with duration of symptoms (from April to June) obtained by clinical history. To be included in the study, a monosensitization to grass pollen had to be demonstrated, based on negative results to SPT with any other aeroallergen. All patients were clinically classified according to ARIA guidelines for AR [14] and to the presence or absence of asthma, as assessed by a physician diagnosis based on clinical symptoms of the disease, as suggested by the Global Initiative on Asthma (GINA) guidelines [15].

The study was conducted in accordance with the Helsinki Declaration and the rules of Good Clinical Practice. Because the study did not use experimental procedures, the local Ethical Committee (Hospital Guglielmo da Saliceto, Piacenza, Italy) was simply informed about the conduction of the observational study and the planned publication of data.

sIgE to Phl p 1, Phl p 5, Phl p 7, and Phl p 12 from *P. pratense* were measured using the CAP system (Thermofisher Phadia, Uppsala, Sweden). The correlation between the ARIA stage of AR and the presence of asthma was analyzed using the Spearman Rank test; the correlation between the clinical data and the levels of sIgE to single components was analyzed by multivariate logistic regression concerning IgE and ARIA stage, while univariate logistic regression was used for IgE and asthma due to the dichotomic classification of asthma as present or absent.

## Results

**Figure 1:**
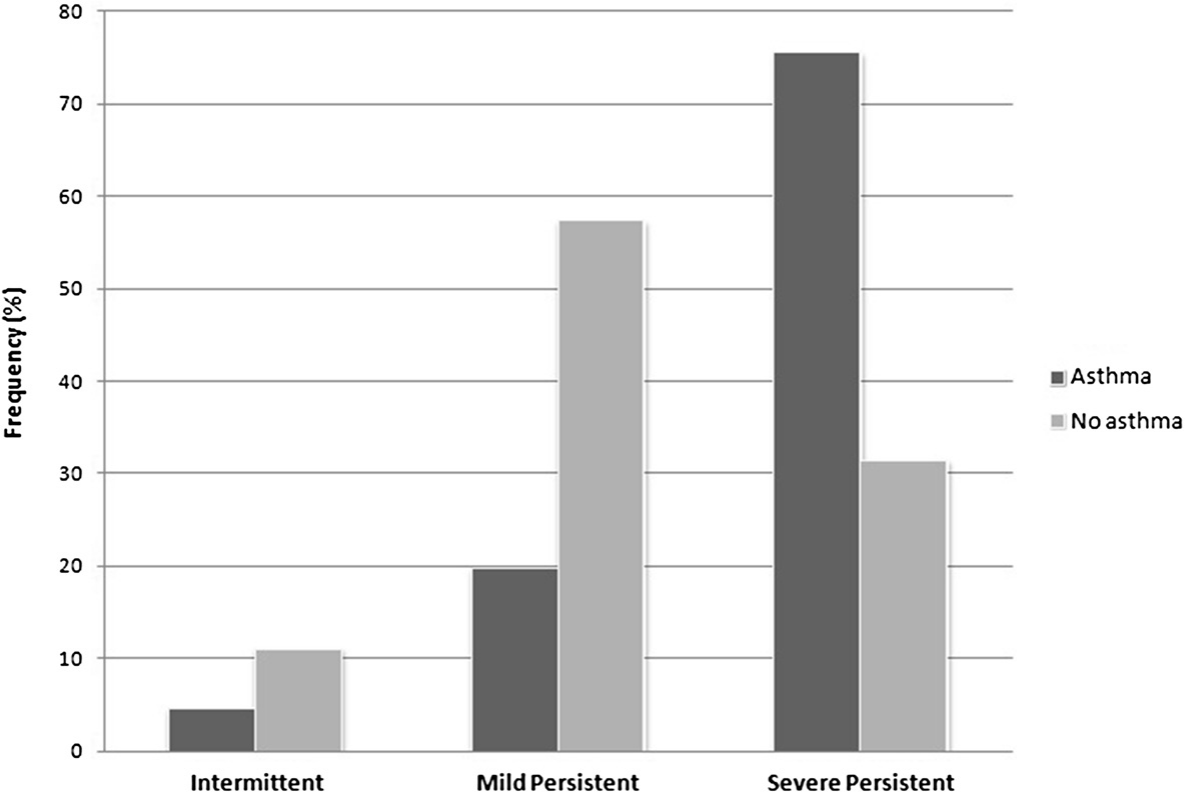
Presence/absence of asthma according to ARIA classification.

**Figure 2:**
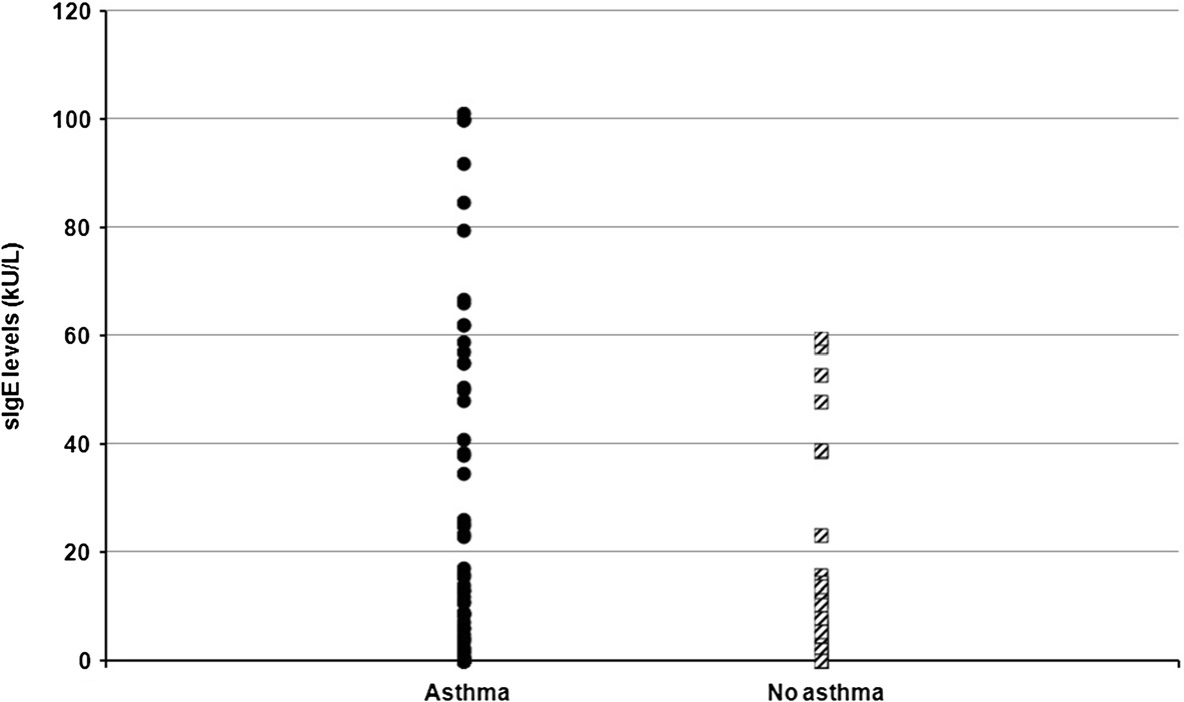
Specific IgE to Phl p 5 in patients with and without asthma.

Of 140 patients (71 males, 69 females, mean age: 29.59 ± 14.68 years), 86 suffered from asthma and 16% were smokers. Concerning the clinical classification of rhinitis according to the ARIA guidelines, 10 patients had intermittent, 48 had mild persistent and 82 had severe persistent AR. A significant correlation was found between severe persistent AR and presence of asthma (*p* < 0.01) (Figure 1). The mean level (SD) of sIgE to the single allergen components were 23.41 (29.46) kU/L for Phl p 1, 16.04 (25.57) for Phl p 5, 0.55 (2.42) for Phl p 7, and 3.04 (8.46) for Phl p 12. No correlation was observed between the levels of sIgE to Phl p 1, Phl p 5, Phl p 7 and Phl p 12 and the ARIA classification of severity. The only significant correlation between clinical data and sIgE values was that of the low values of sIgE to Phl p 5 and absence of asthma (Figure 2).

## Discussion

CRD, based on molecular allergology and biochip technology, allows a comprehensive analysis of individual sensitization profiles with multiplexed or monoplexed purified or recombinant allergens, providing much more information than do allergy tests based on extracts containing a mixture of all allergens [3]. The use of CRD as a tool to prescribe a more precise AIT has recently emerged. In fact, AIT is the only treatment of allergy targeting the causes of the disease and not only its symptoms, which instead are the aim of drug treatment [16], and increased precision is likely to be mirrored by increasing efficacy. In particular, detecting the primary sensitizing allergens and distinguishing them from ubiquitous, cross-reacting allergen (pan-allergens) of poor clinical significance significantly helps in choosing the correct AIT in patients with apparent multiple sensitization [17]. For grass pollen, pan-allergens are represented by polcalcins (pollen calcium-binding proteins), corresponding to Phl p 7 and its homologous molecules [18], and profilins, corresponding to Phl p 12 and its homologous molecules [19]. On the other hand, Phl p 1 and Phl 5 are primary sensitizing allergens. Recent studies on patients with grass pollen allergy proposed models of interpretation based on different combinations of the IgE response to the array of allergen molecules [13]. By examining 200 children with grass pollen-induced AR, asthma, or both, Tripodi et al. investigated the profiles of IgE sensitization to *P. pratense* to define the compatibility of these profiles with a mixture of recombinant allergenic molecules of *P. pratense* previously proposed for AIT. Patients sera were tested for the individual molecules (rPhl p 1, rPhl p 2, rPhl p 4, nPhl p 4, rPhl p 5b, rPhl p 6, rPhl p 7, rPhl p 11, and Phl p 12) and the individual IgE sensitization profiles were matched to the AIT preparation containing Phl p 1, Phl p 2, Phl p 5, and Phl p 6; 39 profiles of sensitization were identified, and the molecular profile of the AIT preparation matched that of 4% of patients only; the remaining patients were classified in four mismatch categories: underpowered (29%), overpowered (32%), underpowered/overpowered (32%), and unrelated (3%). The authors concluded that IgE sensitization profiles to *P. pratense* are highly heterogeneous and that molecularly designed AIT preparations tailored to patients’ needs should consider this high heterogeneity. However, this model is far too complex to be used in current practice when deciding the appropriate AIT for single patients, and can be further confounded if extracts containing multiple grasses, and not only *P. pratense*, are used [20]. In addition, the recombinant allergen preparation that poorly fit with the study patients in fact showed excellent clinical results in a previous controlled trial [21]. In the present study, we limited the investigation to the main primary sensitizing molecules (Phl p 1 and Phl p 5) and to the main cross-reacting molecules (Phl p 7 and Phl p 12), with the aim to exploring the possible correlation between the patient’s IgE response to such allergens and clinical data. Clinically, an association between a more severe AR and occurrence of asthma was found, confirming previous observations [22]. However, no correlation was seen between the ARIA stage of rhinitis and sIgE levels to single allergen components. To our knowledge, this is the first study evaluating such relationship. Previous studies measuring sIgE levels to the whole pollen reported a lack of correlation between titers of serum allergen-specific IgE to grass pollen and rhinitis symptoms [23], while such a correlation was found in patients allergic to mugwort and ragweed [24]. Indeed, the significant correlation we detected between absence of asthma and low values of sIgE to Phl p 5 deserves to be tested for clinical application.

## Conclusions

The finding of this study suggest a possible use of low values of sIgE to Phl p 5 as surrogate marker when selecting patients who are candidates for AIT, since a higher risk of asthma in patients with high levels of sIgE to Phl p 5 could be used as a selection criterion for performing AIT. However, such observations need to be confirmed in further studies before being applied in current clinical practice.

## Abbreviations

AIT: Allergen immunotherapy; AR: Allergic rhinitis; CRD: Component resolved diagnosis; sIgE: Specific IgE.

## Competing interests

Ilaria Dell’Albani and Franco Frati are employees of Stallergenes Italy. Cristoforo Incorvaia and Francesco Marcucci are scientific consultants for Stallergenes Italy.

## Authors’ contributions

ES Designed the study and analyzed the results. SP Performed the laboratory analysis and collected the data. CI Analyzed the results and drafted the manuscript. ID Analyzed the results and drafted the manuscript. FM Analyzed the results and drafted the manuscript. GDC Performed the statistical analysis. FF Designed the study, analyzed the results and drafted the manuscript. All authors read and approved the final manuscript.
